# Comparative Perspectives on Geriatrics-Surgery Co-Management Program by Specialty and Staff Role

**DOI:** 10.1093/geroni/igab046.1260

**Published:** 2021-12-17

**Authors:** Sonia Pandit, Mark Simone, Alyson Michener, Lisa Walke, Ingrid Nembhard

**Affiliations:** 1 University of Pennsylvania, Philadelphia, Pennsylvania, United States; 2 University of Pennsylvania, University of Pennsylvania, Pennsylvania, United States; 3 The Wharton School, University of Pennsylvania, Philadelphia, Pennsylvania, United States

## Abstract

Co-management programs between geriatrics and surgical specialties have gained popularity in the last few years. Little is known about how these programs are perceived across surgical specialties and staff roles. We conducted a mixed methods study to assess perspectives on a geriatrics-surgery co-management program (GSCP) at a hospital where geriatricians co-manage patients 65 or older admitted to Orthopedic Trauma, General Trauma, and Neurosurgery. We used semi-structured interviews (n=13) and online surveys (n=45) to explore program value, facilitators, use, understanding, and impact by specialty and staff roles (physicians, advanced practice providers, nurses, case managers, social workers). Interview transcripts were analyzed using qualitative thematic analysis, and survey data were analyzed using Kruskal-Wallis, ANOVA, and Fisher’s exact tests. Interviews revealed three themes: 1) GSCP is valued because of geriatricians’ expertise in older adults, relationship with patients and families, and skill in addressing social determinants of health; 2) GSCP facilitators include consistent availability of geriatricians, clear communication, and collaboration via shared data-driven goals; and 3) GSCP use varies by surgical specialty and role depending on expertise and patient complexity. Survey data analysis affirmed interview themes and showed significant differences (p-values<0.05) between perspectives of surgical specialties and roles on GSCP use, understanding, impact, and which specialty should manage specific clinical issues. Findings suggest that while there are similarities across surgical specialties and roles regarding the value of, and facilitators for, a GSCP, specialties and roles differ in use, understanding, and perceived program impact on care. These findings suggest strategies for optimizing this intervention across groups.

